# Genetics of migraine aura: an update

**DOI:** 10.1186/s10194-020-01125-2

**Published:** 2020-06-05

**Authors:** Irene de Boer, Gisela M. Terwindt, Arn M. J. M. van den Maagdenberg

**Affiliations:** 1 Department of Neurology, Leiden University Medical Center, Albinusdreef 2, PO Box 9600, 2300 RC Leiden, The Netherlands; 2 Department of Human Genetics, Leiden University Medical Center, Albinusdreef 2, PO Box 9600, 2300 RC Leiden, The Netherlands

**Keywords:** Migraine, Aura, Genetics, GWAS, Complex, Variant, Mutation, Monogenic

## Abstract

Migraine is a common brain disorder with a large genetic component. Of the two main migraine types, migraine with aura and migraine without aura, the genetic underpinning in the former is least understood. Given the evidence from epidemiological studies in cohorts and families that the genetic contribution is highest in migraine with aura, this seems paradoxical. Various genetic approaches have been applied to identify genetic factors that confer risk for migraine. Initially, so-called candidate gene associations studies (CGAS) have been performed that test DNA variants in genes prioritized based on presumed a priori knowledge of migraine pathophysiology. More recently, genome-wide association studies (GWAS) tested variants in any gene in an hypothesis-free manner. Whereas GWAS in migraine without aura, or the more general diagnosis migraine have already identified dozens of gene variants, the specific hunt for gene variants in migraine with aura has been disappointing. The only GWAS specifically investigating migraine with aura yielded only one single associated single nucleotide polymorphism (SNP), near *MTDH* and *PGCP*, with genome-wide significance. However, interrogation of all genotyped SNPs, so beyond this one significant hit, was more successful and led to the notion that migraine with aura and migraine without aura are genetically more alike than different. Until now, most relevant genetic discoveries related to migraine with aura came from investigating monogenetic syndromes with migraine aura as a prominent phenotype (i.e. FHM, CADASIL and FASPS). This review will highlight the genetic findings relevant to migraine with aura.

## Background

Migraine is a common brain disorder characterised by attacks of severe typically unilateral headache with nausea, vomiting, phono- and/or photophobia [1]. In up to a third of patients, attacks are accompanied by an aura, consisting of transient focal neurological symptoms that are most often visual, but may also include sensory symptoms and/or speech disturbances. If the aura encompasses motor weakness the disease is diagnosed as hemiplegic migraine [1]. The likely underlying mechanism of the aura is cortical spreading depolarisation (CSD), a brief wave of neuronal and glial depolarization that slowly self-propagates across the cerebral cortex, originating in the occipital cortex, that is followed by long-lasting suppression of brain activity [2–4].

Migraine has a strong genetic component. Epidemiological studies have shown that migraine runs in families and that migraine concordance rates are 1.5- to 2-fold higher in monozygotic compared to dizygotic twins [5–7]. A stronger family history of migraine is associated with migraine with aura, as well as a lower age-at-onset and a higher number of medication days [8], which suggests a higher genetic susceptibility in this migraine type [8]. Several approaches, discussed below, have been used to identify genetic factors that confer migraine risk. The general assumption is that in common polygenic forms of migraine multiple DNA variants, each with a small effect size, together with environmental factors confer disease risk, whereas in rare monogenic migraine syndromes a single DNA mutation suffices to cause disease in a patient.

### Candidate gene association studies in migraine with aura

Initially, candidate gene association studies (CGAS) were performed that test whether specific genetic markers in genes that had been selected based on prior hypotheses that they are implicated in migraine disease pathways, show a different allele frequency in migraine cases vs. controls. CGAS have been performed for many candidate genes for both migraine types (for reviews see [9, 10]). Among the largest studies is one that included 841 migraine with aura patients and 884 controls in the discovery cohort and tested several thousand genetic markers in 155 ion transporter genes, but replication in an independent data set was essentially negative [11]. A large CGAS that systematically re-evaluated association findings of the 21 most promising genes from CGAS, but now using a much larger data set of several thousand cases and controls, did not lead to significant results [12]. Hence one may draw the conclusion that many, if not all, of the initial CGAS results may be false-positives. Results are disappointing most likely because of methodological issues, foremost because, almost without exception, CGAS had too small sample sizes (in most cases less than a few hundred cases and an equal number of controls were tested). Another likely explanation is that the diagnosis of aura symptomatology is difficult to obtain through questionnaires and is often not validated through direct interviews by trained headache physicians [13, 14]. In addition, cases and controls may not have been adequately matched for genetic background, gender and age. Finally, without replication of the main results in an independent population in the same study, the risk of reporting false positive results is high. It remains to be tested whether rarer variants (instead of the more common variants usually tested in a CGAS) may in fact elevate risk in specific subgroups of migraine patients. There is also the distinct possibility that the selected genes are not those that confer genetic risk, even if they belong to molecular pathways clearly implicated in migraine pathophysiology (such as genes that play a role in serotonin and dopamine pathways). Perhaps genes regulating (in)directly these pathways would have been better candidates.

### Genome-wide association study in migraine with aura

Due to improved technology and the development of cost-effective genotyping platforms genome-wide association studies (GWAS) became feasible in the last decade. Unlike CGAS, in a GWAS no prior hypothesis is formulated about the possible role of a DNA variant. In a GWAS, in one experiment, several hundred thousand to millions of single nucleotide polymorphisms (SNPs), close to equally distributed over the whole genome, are tested for association with a trait (e.g. a disease such as migraine) by assessing differences in allele frequencies between patients and controls. Findings are rigorously corrected for the vast number of tests as an association is considered genome-wide significant only if the p-vale is < 5 × 10^− 8^. In a GWAS typically only variants with a moderate to high minor allele frequency (≥ 0.05) are genotyped; so alternative approaches, foremost next generation sequencing (NGS)-based whole-exome and whole-genome approaches, are used to assess the contribution of rarer variants.

The first migraine GWAS consisted of 2731 patients with migraine with aura, obtained from three European specialized headache clinics (from Finland, Germany and the Netherlands), and 10,747 population-matched controls in the discovery cohort [15]. Surprisingly, only one single SNP, rs835740, reached genome-wide significance in this sample (*p* = 5.38 × 10^− 9^, odds ratio = 1.23). The finding was successfully replicated in an independent set of samples from Iceland, Denmark, Germany and the Netherlands encompassing 3202 cases and 40,062 controls and was highly significant in an overall meta-analysis (*p* = 1.69 × 10^− 11^, odds ratio = 1.18). The associated SNP is located between *MTDH* (better known as astrocyte elevated gene 1 (*AEG-1)*) and *PGCP* (encoding plasma glutamate carboxypeptidase); both gene products have been implicated in glutamate homeostasis. A subsequent analysis of gene expression in lymphoblastoid cell lines demonstrated that the risk allele correlated with a higher transcript level of the *MTDH* gene [15]. However, the relation between *MTDH* and migraine remains controversial, also because the SNP did not show significance in subsequent meta-analyses of GWAS data, neither in studies that investigated association with migraine (*p* = 5 × 10^− 3^ [16], *p* = 0.12 [17]) nor migraine with aura (both when the cohorts used to identify the locus were included (*p* = 1.49 × 10^− 4^) or excluded (*p* = 0.59) [17]).

The most recent migraine GWAS included 59,674 cases and 316,078 controls and identified up to 38 distinct genomic regions associated with migraine [16]. When considering the genes closest to the associated SNPs, which is the typical way SNPs are being linked to implicated genes, it was concluded that current GWAS findings seem to link to neuronal, vascular, metalloproteinase, pain and metal-iron-related pathways [16–18]. Only in sub-analyses the contribution of associated loci to specific migraine types was given further consideration. Whereas seven loci (i.e. near *TSPAN2*, *TRPM8*, *PHACTR1*, *FHL5*, *ASTN2*, near *FGF6*, and *LRP1*) were associated with migraine without aura (in a sample of 8348 cases and 139,622 controls), none were associated with migraine with aura (6332 cases vs. 144,883 controls) [16]. Of note, the seven loci had already been identified in the initial analysis with migraine in general, not subdivided in its subtypes, as phenotype. Given that two-third of migraineurs suffers from migraine without aura, this finding is perhaps not that surprising as it merely shows that the most abundant migraine type likely seems responsible for the association signals.

### Is the genetic component in migraine with and without aura different?

A possible explanation for why GWAS appears more successful in migraine without aura might be that both migraine types are distinct disorders with a different genetic architecture. The suggestion fuels the long-standing debate whether or not both types are different disease entities. It should be noted that the migraine types are diagnosed differently according to the ICHD-III classification as migraine with aura, unlike migraine without aura, does *not* have to fulfil two out of four headache characteristics (moderate/severe pain intensity, unilateral location, pulsating quality, aggravation by routine physical activity) and accompanying symptoms (photophobia and phonophobia and/or nausea or vomiting). Moreover, to be labeled as patient the experienced number of attacks is less in migraine with aura (at least two attacks) compared with migraine without aura (at least five attacks) [1]. Notwithstanding, there are long-recognized clinical observations suggestive for a shared etiology of both migraine types, not only because attacks of both types can co occur in a patient (or family) or that the migraine type can change over lifetime (aura attacks may develop in the elderly patient) [19].

It was the hope that GWAS findings could settle the debate. One argument for the distinction of migraine types is the previously mentioned observation that GWAS particularly identified loci for migraine without aura. Another genetic argument is that a polygenetic risk score analysis of 21 migraine-associated SNPs from GWAS showed association only with migraine without aura [20]. Importantly, it should be noted that in that study participants with migraine with aura (152 cases) were fewer than participants with migraine without aura (295 cases), so the study likely lacks statistical power to pick up a signal for migraine with aura. A recent study that took into account all available genetic information, so not just the migraine-associated SNPs (‘top hits’), in a much larger data set, however, found statistical evidence for a significant overlap of genes associated with migraine with aura (4505 cases) and migraine without aura (4038 cases) and concluded that the genetic architecture of both types is more alike than dissimilar [21]. Likewise, a heterogeneity analysis of SNP effects in migraine with aura and migraine without aura showed that for 12 migraine-associated SNPs the risk-increasing allele was the same [22]. In fact, when more than 23,000 SNPs were analyzed the majority of SNP effects were in the same direction in migraine with aura (5118 cases) and migraine without aura (7101 cases) [22]. Finally, the international Brainstorm consortium that compared GWAS data of over 20 brain disorders (and investigated genetic information of 265,218 patients and 784,643 controls) found substantial genetic correlation between migraine with aura and migraine without aura [23]. In conclusion, although migraine with aura and migraine without aura seem genetically more alike than dissimilar, the challenge remains to identify more of the underlying causal variants as only a fraction of them have been discovered.

### Where is the missing heritability of migraine with aura?

**Fig. 1:**
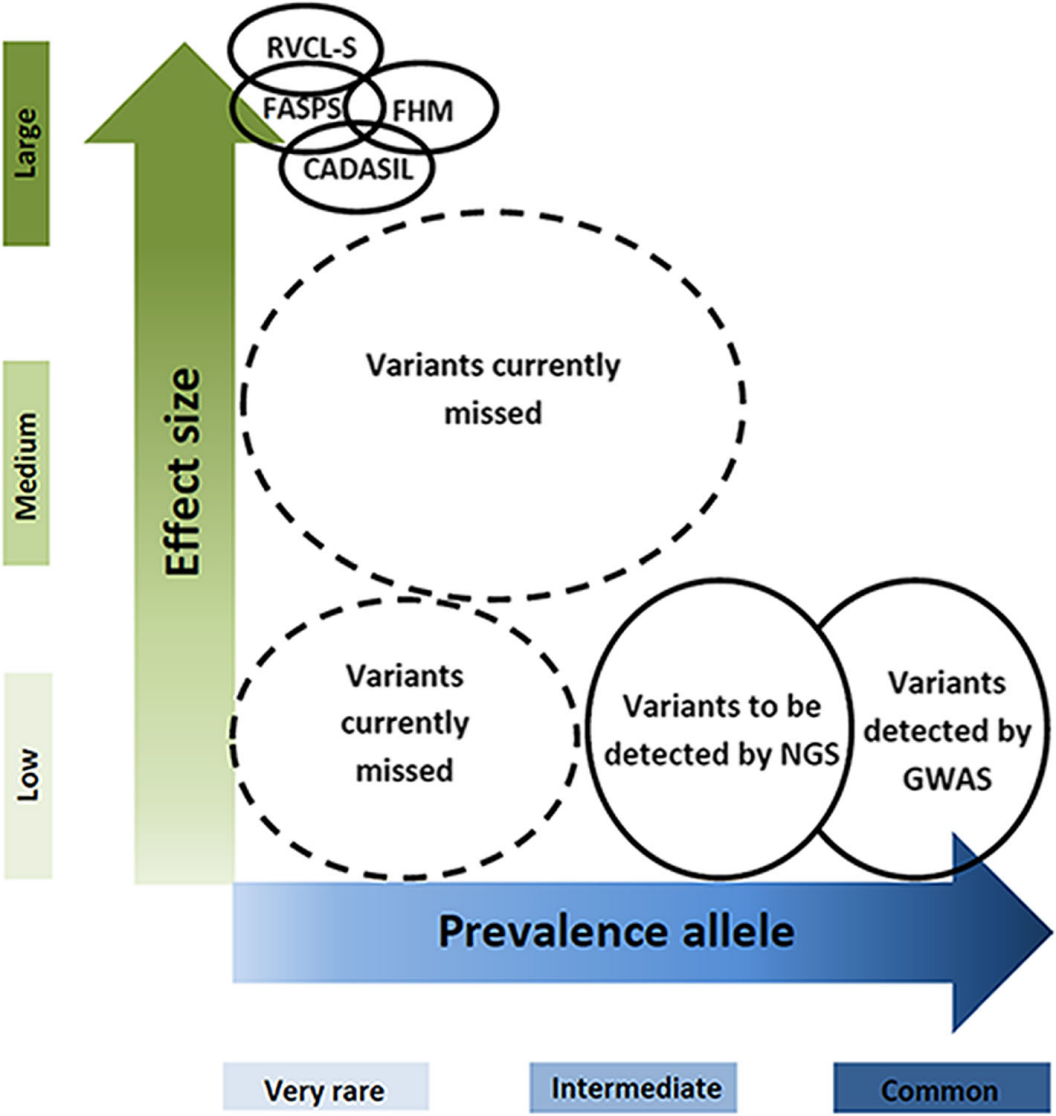
Missing genetic component of migraine with aura. CADASIL: cerebral autosomal dominant arteriopathy with subcortical infarcts and leukoencephalopathy, FASPS: familial advanced sleep-phase syndrome, FHM: familial hemiplegic migraine, GWAS: genome-wide association studies, NGS: next generation sequencing, RVCL-S: retinal vasculopathy with cerebral leukoencephalopathy and systemic manifestations

Like what has been proposed for other complex diseases, one can postulate that migraine with aura may be determined by multiple rarer medium- to high-risk alleles that are not captured by a GWAS approach [24] (Fig. 1). Finding an association for rarer alleles is notoriously difficult as it requires the analysis of even larger well-defined migraine with aura cohorts and representation of rarer variants in SNP arrays. For rarer variants a more logical approach is to genotype them by NGS, which has not been done yet.

Part of the missing heritability could also be explained by genetic variants in mitochondrial DNA (mtDNA). Mitochondrial DNA is separated from nuclear DNA and inherited exclusively along the maternal lineage. Each cell contains hundreds to thousands of copies of mtDNA, and there may be genetic variation between mtDNA molecules [25]. In fact, migraine-like headache has been reported in several mitochondrial disorders, such as mitochondrial encephalopathy, lactic acidosis and stroke-like episodes (MELAS) [26]. Additionally, structurally abnormal mitochondria and findings indicative of impaired energy metabolism have been demonstrated in migraine [27]. While small CGAS focusing on mtDNA have reported associations [28], these could not be replicated in a recent mitochondrial GWAS (with 4021 migraine cases included) [29]. Furthermore, this mitochondrial GWAS found no additional associations with migraine [29]. It seems likely that a larger sample size is required to find variants robustly associated with migraine.

Another explanation for the missing heritability could be epigenetic processes. Epigenetics encompasses changes to the DNA structure without changing the genetic code and affects gene expression and has also been implicated in neurological disorders [30]. Epigenetic changes can be dynamic, be passed on through cell divisions or transgenerational inheritance. The main epigenetic modifications are DNA methylation and post-translational modification of histone proteins. So far, small steps have been taken to dissect the epigenetic contribution towards migraine [31]. The first genome-wide analysis of DNA methylation in migraine identified 62 independent differentially methylated regions in blood samples without distinguishing between migraine without and with aura [32]. While epigenetic factors might contribute towards migraine pathophysiology, for instance towards migraine chronification, this field is still in its infancy and therefore it is too soon to speculate towards its relevance for migraine with aura.

### Genes in monogenetic migraine with aura syndromes

Until now successful identification of genetic factors relevant to migraine with aura almost exclusively came from investigating rare monogenetic syndromes. The identification of causal genes for such syndromes is more straightforward than for polygenic disorders, already because the hunt is for a single genetic factor that is sufficient to cause disease in a patient (Fig. 1). Sequencing of the coding regions of the DNA of a patient (and family members) by direct sequencing of exons of possible candidate genes, in the past, and nowadays by sequencing all coding regions in a single experiment by whole exome sequencing has led to the identifications of thousands of disease genes for many disorders [33]; among which a number for migraine with aura syndromes that will be discussed below. Whole exome sequencing is more and more ready to be used in the diagnostic process of migraine with aura syndromes, as shown in a large Finnish sample [34].

### Familial hemiplegic migraine

The most investigated monogenetic migraine with aura syndrome is hemiplegic migraine [1]. In patients with hemiplegic migraine, the auras include motor weakness in addition to other focal neurological features commonly seen in migraine with aura patients. Additional clinical symptoms in hemiplegic migraine may include cerebellar ataxia, seizures, cognitive impairment, and decreased consciousness [35]. Of note, many patients also suffer from attacks of migraine with aura and/or migraine without aura [36]. This finding, but also the observation that factors that trigger attacks are the same as for migraine with aura [37], supports the hypothesis that hemiplegic migraine is part of the spectrum of migraine disorders. DNA sequencing of candidate genes in genomic regions shared by affected family members, have identified causal mutations in three hemiplegic migraine genes: *CACNA1A* (FHM1), *ATP1A2* (FHM2), *SCN1A* (FHM3) [38]. In recent years, whole genome/whole exon NGS was used to identify additional causal genes in those patients in which no mutation in one of the three genes had been found. Surprisingly, this has not led to undisputed additional genes [38]. One important explanation may be that there is clinical heterogeneity among patients with a hemiplegic migraine diagnosis [38]. Chances of finding a causal mutation in hemiplegic migraine patients in one of the known genes is higher in patients with a lower age of disease onset, when there are more affected family members, and when attacks are (1) triggered by mild head trauma, (2) include confusion, (3) have extensive motor weakness, (4) have symptoms of brainstem pathology, or (5) are associated with brain edema; mental retardation and progressive ataxia are only found in hemiplegic migraine patients with a causal mutation in one of the three known genes.

### Other monogenetic migraine with aura syndromes

In patients with monogenic cerebral small vessel diseases a high prevalence of migraine with aura can be observed. Almost half of the patients with cerebral autosomal dominant arteriopathy with subcortical infarcts and leukoencephalopathy (CADASIL) suffer from migraine with aura, which is often the first symptom of disease [39]. Other symptoms of CADASIL include mid-adult onset of recurrent ischemic stroke, cognitive decline progressing to dementia, mood disturbances and apathy. CADASIL is caused by mutations, typically affecting the number of cysteines, in Notch3 that plays an important role in vascular smooth muscle cell functioning [40, 41]. Another small vessel disease in which migraine with (and without) aura is often present is retinal vasculopathy with cerebral leukoencephalopathy and systemic manifestations (RVCL-S) [42, 43]. RVCL-S is caused by carboxyl terminal truncating mutations in TREX1 [44]. In addition to migraine (with and without aura), mutation carriers typically suffer from retinal vasculopathy, focal and global neurological symptoms and systemic manifestations including impaired liver and renal function [42–44]. In RVCL-S migraine onset appears later in life (> 40 years) [43, 45]. This might indicate that migraine is a secondary phenomenon due to the progressive cerebral vasculopathy seen in patients with RVCL-S. Of note, in another cerebral small vessel disease, Dutch-type hereditary cerebral amyloid angiopathy (D-CAA) that is caused by a single point mutation (E693Q) in the amyloid precursor protein (*APP*) gene, there also is an increased prevalence of migraine with aura [46]. D-CAA is characterized by intracerebral hemorrhages and vascular dementia [47]. Migraine with aura is the presenting symptom in close to 80% of patients [46]. Patients with specific mutations in *COL4A1* also appear to have an increased prevalence of migraine with aura, in addition to clinical features such as porencephaly type 1, brain small vessel disease with or without ocular anomalies, non-syndromic autosomal dominant congenital cataract, hereditary angiopathy with nephropathy, aneurysms, muscle cramps syndrome, and/or tortuosity of retinal arteries [48–50]. At present it is not known what causes the higher prevalence of migraine with aura in these genetic vasculopathies. Possible suggested mechanisms are neurovascular changes, including endothelial and vascular smooth muscle dysfunction and amyloid depositions [40, 51–56].

Also in patients with non-small vessel disease familial advanced sleep phase syndrome (FASPS), which is caused by specific missense mutations in the *CSNK1D* gene, migraine with aura is prevalent [57]. Although little over a handful of mutation carriers in two FASPS families have been reported, almost without exception they also suffer from migraine with aura [57]. Given the fact that FASPS is characterized by a profound phase advancement of the sleep-wake, melatonin and temperature rhythms [57–59], other genes involved in sleep regulation might be implicated in familial migraine. A good candidate is *PER2*, which is already a known cause of FASPS [60]; it is however unknown whether *PER2* mutation carriers also have an increased risk for migraine.

### Other genes implicated in migraine with aura syndromes?

In recent years several genes have been put forward as possible hemiplegic migraine genes (i.e. *PRRT2, SLC1A3* and *SLC4A4* [61–65]), but evaluation of available data should cast doubt on such claims.

*PRRT2* - Whereas heterozygous *PRRT2* mutations (typically involving similar deletions in the same cytosine stretch) have been identified in a few patients with hemiplegic migraine [63, 64, 66, 67], it must be taken into account that many hundreds of patients with such mutation who suffer from paroxysmal kinesigenic dyskinesia, benign familial infantile seizures, or infantile convulsion choreoathetosis syndrome [66] do *not* have hemiplegic migraine. So the presence of such *PRRT2* mutation does not suffice to cause hemiplegic migraine in the Mendelian fashion seen with the three hemiplegic migraine genes [64]. Thus, *a PRRT2* mutation may increase the risk of hemiplegic migraine as “add-on” mutation but it seems unlikely that PRRT2 is the FHM4 gene. Still PRRT2 itself is relevant given functional evidence that mutant PRRT2, which affects the neuronal neurotransmitter release machinery, leads to abnormal neuronal hyperexcitability, as was shown in a knockout rat model [68].

*SLC1A3* - The observation of a missense mutation P290R in *SLC1A3 *in a single patient with alternating hemiplegia, episodic ataxia, and seizures, does not qualify *SLC1A3* as a bona fide hemiplegic migraine gene either [62]. *SLC1A3* encodes the excitatory amino acid transporter 1 (EAAT1) that is involved in glutamatergic neurotransmission. More recently, *SLC1A3* missense mutation T387P, shown to diminish potassium binding to EAAT1, was identified in a patient with hemiplegic migraine, strengthening the believe that EAAT1 might be involved in hemiplegic migraine. Still, doubts concerning causality remains as the mutation did not segregate with the phenotype as the patient’s father was a mutation carrier but did not suffer from hemiplegic migraine [65]. At this moment proof for causality based on isolated cases in which the clinical diagnosis is not firmly established or in which the mutation does not segregate in the family seems not sufficient [62, 65].

*SLC4A4 -* In two patients with proximal renal tubular acidosis and a homozygous frameshift mutation S982NfsX4 in *SLC4A4*, which encodes for a sodium bicarbonate cotransporter NBCe1, hemiplegic migraine was reported, while some heterozygous carriers reported common forms of migraine [61]. Given the notion that NBCe1 dysfunction affects pH and the functioning of astrocytes, the possible involvement of *SLC4A4* in migraine pathophysiology deserves further investigation.

Of note, none of the mutations described in *SLC1A3* and *SLC4A4* above are found in gnomAD, a sequence variant reference database containing the information of over 100,000 exomes [69]. Absence in gnomAD indicates that the mutations must be rare and underscores that the mutations may indeed cause disease albeit that the association with migraine needs more proof.

In addition to the potentially (hemiplegic) migraine genes mentioned above, there is also an interesting report on a gene, *KCNK18*, that was identified in a large multigenerational pedigree suffering from migraine with aura [70]. Thus far, genetic evidence that specific frameshift mutations (such as F139WfsX24) in the *KCNK18* gene, encoding the TRESK channel, cause migraine came from only one *single* family in which the TRESK mutation seemed to segregate perfectly with the phenotype. It was also not reported whether there were other mutations, in the area of linkage, that could equally be disease gene candidates. This scenario is not that unlikely as only a set of 150 ion transporter genes (in the entire genome) were screened for mutations in a set of 110 unrelated migraine patients in the original study [71]. Moreover, loss of one functional copy of TRESK *per se* does not seem to lead to disease as another variant, C110R, was found in both controls and patients [70]. Where functional analyses of cells expressing the frameshift mutation indicated effect on plasma membrane localization leading to hyperexcitability of trigeminal ganglion neurons [72], this second mutation (C110R) did not increase the excitability of trigeminal ganglion neurons [73, 74]. Recent intriguing work sheds some light on the complex mechanism on how TRESK mutations could lead to different functional effects, as frameshift mutations F139WfsX24 and Y121LfsX44, but not C110R, lead to an alternative start codon and consequently production of a second TRESK protein, which co-assembles with TREK1 and TREK2 to downregulate their function leading to increased trigeminal ganglion neuron excitability [75]. This might be the reason why only specific frameshift mutations induce hyperexcitability in trigeminal ganglion neurons. Using CRISPR-Cas9 engineering to correct the F139WfsX24 mutation in induced pluripotent stem cell-based models, a reversal of the neuronal excitability phenotype was demonstrated [76].

Why shed doubt whether TRESK1 mutations are indeed causal for migraine? Firstly, proof of causality based on one single family, even though segregation is seemingly perfect, is not sufficient, especially when realizing that the linkage region was not thoroughly scanned for other possible causal mutations. Secondly, whereas another frameshift mutation Y121LfsX44 was reported in ClinVar [77] (RCV000490385.1) to be associated with migraine (notably without any record clinical details) [75], for a second case no association with disease was reported. Thirdly, and foremost, frameshift mutations in *KCNK18* in gnomAD are among the most common variants in *KCNK18* [69]. The Y121LfsX44 and the F139WfsX24 mutations were found no less than 665 and 123 times, respectively. Although in theory all individuals may have familial migraine, this sheds reasonable doubt on whether TRESK is indeed a migraine gene. The more likely scenario is that TRESK mutations that lead to inactive protein function are well tolerated, which is also suggested by the observation that there are 12 homozygous carriers reported in gnomAD. While it thus seems unlikely that a single mutation in TRESK is sufficient to cause migraine with aura, this does not exclude a role for TRESK in migraine pathophysiology. Based on their mode of action TREK1 and TREK2 might qualify as potentially interesting therapeutic targets for migraine.

### Functional consequences of gene mutations causing migraine with aura syndromes

To unravel the pathophysiological mechanisms causing hemiplegic migraine, functional consequences of gene mutations have been studied in cellular and animal models (for detailed reviews see [78–80]). First clues came from studies in cellular models in which mutant cDNAs were overexpressed. FHM1 mutations in the α1 subunit of voltage-gated Ca_V_2·1 (P/Q-type) calcium channels result in a gain of channel function [81]. FHM2 mutations in the α2 subunit of sodium-potassium ATPases result in a loss of function in glial cells [82]. The functional consequences of FHM3 mutations on Na_V_1.1 channels were at first difficult to assess but recent evidence suggest that they are associated with a gain of channel functioning [83]. Together these findings predict increased neurotransmitter and potassium ion levels in vivo at the synaptic cleft, which facilitates CSD and thereby can explain the aura features in patients with the mutations [82–87].

For all three FHM types knock-in mouse models have been generated by expressing pathogenic human gene mutations in the respective endogenous mouse gene. FHM1 mutant mice with either the R192Q or the S218L mutation reveal increased neuronal calcium influx and enhanced (cortical) excitatory, but not inhibitory, neurotransmitter release [88–91]. FHM2 mutant mice with either the W887R or the G301R mutation showed abnormal glutamate uptake through glial cells [92–94]. For the W887R mutant a reduced rate of glutamate and K^+^ clearance, and a reduced density of GLT-1a glutamate transporters in astrocytic processes surrounding glutamatergic synapses, was shown which facilitated the ignition of experimentally induced CSD [92, 93]. In line with these findings, both the FHM1 and FHM2 mutant mice also demonstrate an increased susceptibility for experimentally induced CSD [90, 92, 95, 96]. Recently, the first FHM3 mouse model (expressing the L263V mutation) was generated that demonstrated, for the first time, *spontaneous* CSD events [97]. Of note, the waves of SD consistently propagated from visual to motor cortex, in line with clinical observations in humans.

To lesser extent, it has been investigated how gene mutations of the non-hemiplegic migraine genes (*NOTCH3*, *TREX1*, *APP*, *COL4A1*, *CSNK1D*) may cause migraine pathophysiology. Notch3 protein plays an important role in vascular smooth muscle cells of the small blood vessels of the brain [41]. *Notch3* knockout mice and mice that overexpress the R90C mutation specifically in vascular smooth muscle cells were found to have an enhanced susceptibility to experimentally induced SD [51]. In mutant mice that express the FASPS CKIδ-T44A mutation in *CSNK1D*, which encodes CK1D a well-known regulator of circadian rhythms, the susceptibility is also enhanced [57]. It was hypothesized that migraine with aura in FASPS is caused by vascular dysfunction through abnormal astrocytic signaling. There have been no studies yet that investigated the susceptibility of CSD in mutant mice with *TREX1*, *APP* or *COL4A1* mutations*.* Regardless, there are indirect clues how mutations in these genes may cause a migraine phenotype. RVCL-S mutations in TREX1, the major 3′-5’exonuclease in mammals, were shown to affect the regulation of oligosaccharyltransferase activity [98], but not the protein’s exonuclease function [44, 98]. Recently, homozygous knock-in mice that express the RVCL-S V235fs mutation were shown to demonstrate signs of abnormal vascular function and increased sensitivity to experimentally induced stroke [52]. Of note, vasculopathy and endothelial dysfunction have also been demonstrated in vivo in patients with RVCL-S [53]. Amyloid precursor protein (APP) also is implicated in regulating endothelial function [54], in addition to synapse formation [55] and neuroplasticity [56]. In APPDutch mice (overexpressing the E693Q *APP* mutation) neuronal expression of APPDutch leads to cerebrovascular amyloidosis, hemorrhage and smooth muscle cell degeneration [54], supportive of the idea that vasculopathy may be implicated in migraine. Finally, *COL4A1*, which encodes the procollagen type IV α1, a basement-membrane protein, causes a pleiotropy of vascular disorders. Mutant mice (expressing the *Col4a1*^Δex40^ mutation) develop a compromised cerebral vasculature (intracellular accumulation of mutant collagen in vascular endothelial cells and pericytes triggers small vessel disease) leading to recurrent hemorrhagic strokes and age-related macroangiopathy [50, 99]. Based on the investigations in the mutant mice, it is well possible that vascular changes, such as endothelial dysfunction, may directly or via neuronal pathways increase migraine susceptibility, although this needs to be further investigated.

### Are hemiplegic migraine and the common forms of migraine part of one genetic spectrum?

Recently, the close link between hemiplegic migraine (in those that do not have a causal mutation in the known genes) and migraine with aura was further demonstrated in a large Finnish study [100] that used polygenic risk scoring, based on genetic information of common variants from the most recent migraine GWAS [16]. It was shown that both familial migraine with aura and hemiplegic migraine cases had higher polygenic risk scores, so to larger extent are explained by the presence of common variants, compared to familial migraine without aura cases. The group of patients with hemiplegic migraine had a higher genetic load than in migraine with (typical) aura but the difference did not reach statistical significance (*p* = 0.09) [100], which could be due to a smaller sample size or because sensory symptoms in some patients with migraine with aura erroneously were mistaken for motor symptoms so these patients ended up in the hemiplegic group. Notwithstanding, the study provides an independent argument that hemiplegic migraine and migraine with aura are part of the migraine spectrum.

## Conclusion

Great effort went into unraveling the genetic architecture of migraine, both of migraine with aura and migraine without aura. Although the CGAS approach has been used in many (often very small) studies, findings were not replicated in a much larger GWAS data set. Of note, when taking all genotype information (not just the SNPs that surpass the threshold for genome-wide significance) into account, the genetic architecture of migraine with aura and migraine without aura is more similar than dissimilar. Until now most knowledge of the genetics of migraine with aura came from studies of monogenic syndromes that pointed to roles for neurotransmission and vasculature in migraine pathophysiology. Finally, the genetic load, i.e. the contribution of common genetic variants in families, was particularly high in hemiplegic migraine and migraine with aura compared to migraine without aura re-emphasizing the existence of a migraine spectrum. By increasing patient cohorts, by testing the contribution or rare(r) variants using recently developed SNP arrays and next generation sequencing approaches and by studying possible epigenetic contributors, it is expected that we will get a better insight of the genetic architecture of migraine, including migraine with aura.

## Data Availability

Not applicable.

## Abbreviations

CADASIL: Cerebral autosomal dominant arteriopathy with subcortical infarcts and leukoencephalopathy
CGAS: Candidate gene associations studies
CSD: Cortical spreading depolarization
D-CAA: Dutch-type hereditary cerebral amyloid angiopathy
GWAS: Genome-wide association studies
FHM: Familial hemiplegic migraine
FASPS: Familial advanced sleep phase syndrome
MELAS: Mitochondrial encephalopathy, lactic acidosis and stroke-like episodes
mtDNA: mitochondrial DNA
NGS: Next generation sequencing
RVCL-S: Retinal vasculopathy with cerebral leukoencephalopathy and systemic manifestations
SHM: Sporadic hemiplegic migraine
SNP: Single-nucleotide polymorphisms
